# Effectiveness of AI-driven Individualized Learning Approach for Children with Autism Spectrum Disorder (ASD)

**DOI:** 10.1192/j.eurpsy.2024.205

**Published:** 2024-08-27

**Authors:** H. Atturu, S. Naraganti

**Affiliations:** ^1^Dept of Psychiatry, CARE Hospitals; ^2^Dept of Clinical Psychology, Rainbow Children’s Hospital, Banjara Hills, Hyderabad, India

## Abstract

**Introduction:**

Autism Spectrum Disorder (ASD) is a condition with varying degrees of social, emotional and behavioural disability. These children require focused and individualised learning plan to facilitate social integration. Robots have been used for this purpose but are not routinely available in several parts of the world. Effective, point of care (POC) digital therapies that can be used anywhere by anyone is the need of the day.

**Objectives:**

To evaluate the effectiveness of Artificial Intelligence (AI) driven individualised learning plans delivered through POC digital platform (CognitiveBotics) for children with ASD.

**Methods:**

After Ethical approval and parental consent, children diagnosed with ASD (Childhood Autism Rating Scale CARS 2) aged 2 years and above were screened for study inclusion and exclusion criteria and enrolled. AI driven individualised learning plan was administered through CognitiveBotics software that could be used on either computer or a tablet. Initially, interactive questions were administered to parents by the AI tool to understand child’s functioning. Based on these, an individualised learning plan was assigned. Each task is delivered using either interactive videos, chatbot and/or animated/AI games. The child’s progress is captured for attention (attempted questions, retries and timeouts) and retention (first time corrects and corrects) continuously. The initial interactive questions administered to parents were repeated to assess child’s progress in real life. Paired ‘t’ test using SPSS version 26 was used to compare initial and final data.

**Results:**

Out of 85 registered children, 41 regularly used the AI tool. Mean age was 43.93 months (range 26 to 72 months). 37 (90.24%) were boys. The baseline mean scores (ranges), were CARS 33.48 (30-39.5); Social quotient 53.4 (27.25-80.32), Developmental quotient 71.35 (45.90-93.33) and IQ 62.34 (36.58-86.83). The base line mean score of parents assessed child function was 115.24 (range 58 to 215). A mean of 15.54 tasks were given (range 5 to 48). At the time of analysis with a mean follow up of 3 months (range 2 to 5 months) the children completed a mean of 10.10 tasks (range 0 to 42). There was significant improvement in child’s learning captured by the AI software based on attention and retention parameters (p = <0.00001). This improvement was also reflected in parent assessed child function (mean 147.15, (59 to 231)) (p = <0.00001). The percentage of improvement in both software captured and parent assessed child function was directly related to the amount of time spent by the child on the software.

**Conclusions:**

AI driven individualised learning approach is effective in teaching skills and promote social integration for children with ASD. Such technology can capture the child’s progress on a day-to-day basis and deliver personalised training.

**Disclosure of Interest:**

None Declared

